# Two regional ventilation–perfusion patterns of lung consolidation assessed by electrical impedance tomography and ultrasound

**DOI:** 10.1186/s13054-022-04235-2

**Published:** 2022-11-17

**Authors:** Na Wang, Huaiwu He, Yun Long, Dawei Liu, Qianling Wang, Jing Jiang, Yuechuan Xue, Siyi Yuan, Yi Chi, Zhanqi Zhao

**Affiliations:** 1 Department of Critical Care Medicine, State Key Laboratory of Complex Severe and Rare Diseases, Peking Union Medical College Hospital, Chinese Academy of Medical Sciences, 1 Shuaifuyuan, Dongcheng District, Beijing, China; 2 Department of Critical Care Medicine, Chongqing General Hospital, No. 118, Xingguang Avenue, Liangjiang New Area, Chongqing, 401147 China; 3 Department of Biomedical Engineering, Fourth Military Medical University, Xi’an, China; 4 Institute of Technical Medicine, Furtwangen University, Villingen-Schwenningen, Germany

The perfusion of lung consolidation regions varies, depending on hypoxic vasoconstriction, obstruction or compression of pulmonary capillaries and unobstructed degree of related pulmonary arteries. The electrical impedance tomography (EIT) could make a rapid assessment of functional ventilation and perfusion in the related lung regions, which was helpful for the broad diagnosis and further treatment [1]. However, EIT could not identify a precise anatomical location or the etiology that causes the change of regional perfusion. Ultrasound had been used to map the bronchial arteries, pulmonary arteries and venous in lung consolidation [2]. In this study, we proposed two typical patterns of regional V-Q matching of lung consolidation by EIT and ultrasound at the bedside.

1. Dead-space pattern (D-pattern) was defined as a severe perfusion defect with mild impaired ventilation that results in a regional dead-space in the related lung quasi-consolidated regions (still some ventilation in these regions). In EIT image, high regional Dead-space% should be identified, whereas in ultrasound, absent or dot-like vascularity should be observed in the quasi-consolidated regions. The potential pathophysiologic mechanism could be obstruction or compression of pulmonary capillaries and unobstructed degree of related pulmonary arteries. Respiratory treatment for patients with D-pattern should not only focus on improving regional ventilation but also restoring pulmonary perfusion.

To illustrate the typical EIT and ultrasound images, a case with D-pattern was present.

A 66-year-old man, who had obstructive pneumonia due to central non-small-cell lung cancer in the right lung, was mechanically ventilated. A lower regional ventilation distribution, pleural effusion and consolidation were found in the right lower lobe (Fig. 1A). Color Doppler found an absent vascularity in the consolidation region of right lung. Moreover, using saline bolus EIT also found the perfusion was poor in the right lung that caused a high dead-space. With aim to improve regional ventilation, an increase of PEEP (from 8cmH_2_O to 14cmH_2_O) and drainage of pleural effusion were used. The high PEEP caused a significant improvement of ventilation but less correction of perfusion defect. The range of consolidated region decreased; however, no vascularity was seen within this consolidated lung at the higher PEEP level. The CTPA implied multiple pulmonary artery stenosis of the upper and lower lobe in the right lung. In this case, some shunt% region present in the left lung without pulmonary artery embolism, which indicated redistribution of lung blood perfusion. Moreover, the V/Q match was impaired in the high PEEP that cause leading to heterogeneity in pulmonary perfusion.

**Fig. 1:**
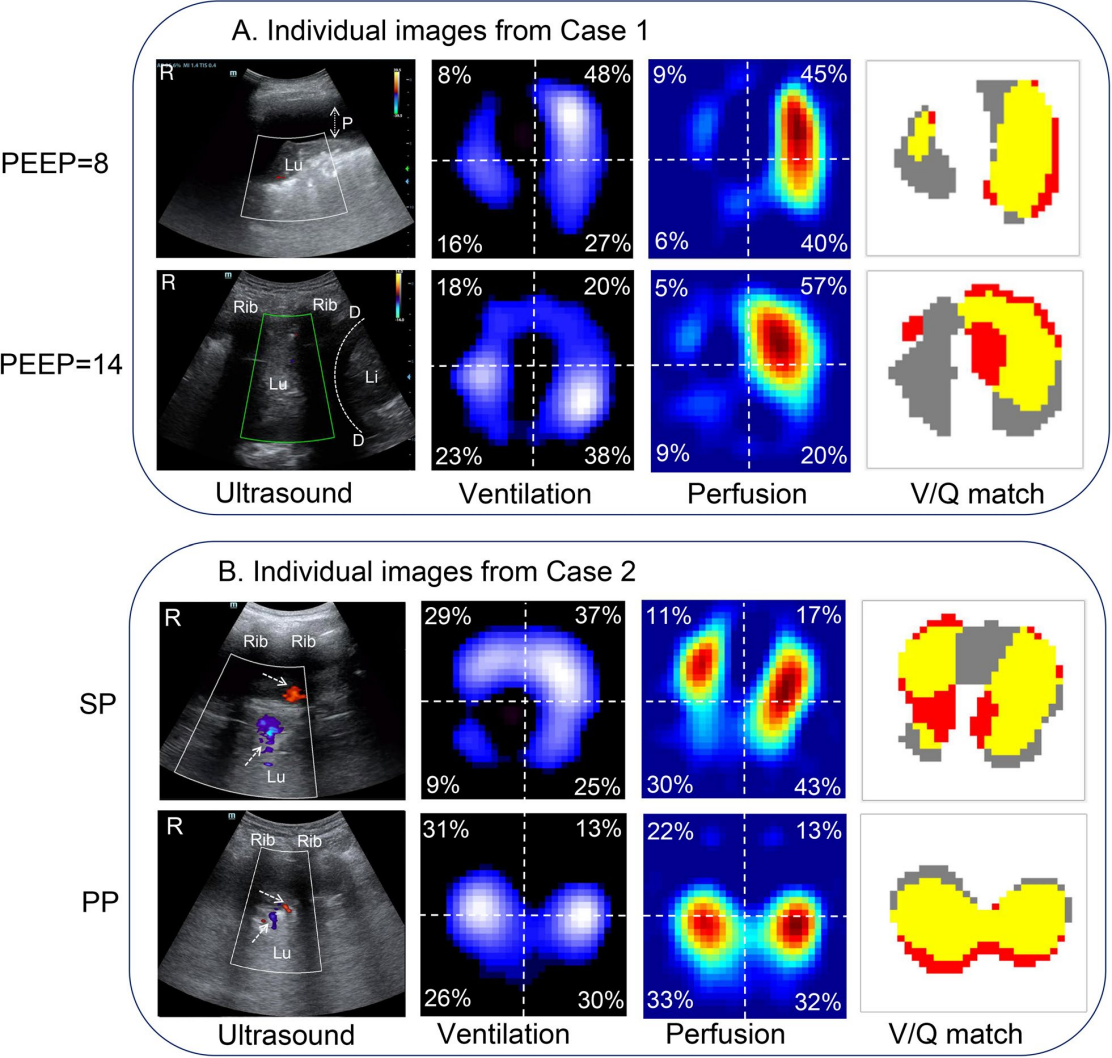
**A** Regional ventilation, perfusion, intrapulmonary shunt and deadspace assessed by lung ultrasound(DC-40S Mindray, Shenzhen, China) and EIT(PulmoVista 500, Dräger Medical, Lübeck, Germany) in a D-pattern case. EIT measurements were continuously recorded at 20 Hz, which were digitally filtered using a low-pass filter with a cutoff frequency of 0.67 Hz to eliminate periodic cardiac-related impedance changes. PEEP = 8 cm H_2_O from left to right, ultrasound: demonstrated pleural effusion and massive consolidation in the right lower lobe, and color Doppler ultrasound found an absent vascularity in the consolidation region. Pleural effusion frequently appears as an echo-free zone between visceral and parietal pleura, of which the distance indicates the depth of pleural effusion(white arrow). Inside the pleural effusion, a floating consolidated lobe could easily be distinguished. The consolidated region appears tissue-like pattern with no aeration. Within consolidated regions, blood flow signals were defined as pulsatile flow shaped of dot, tube, curve, or branch using Doppler with color-flow mapping in the color mode. Absent vascularity was defined as no pulsatile blood flow was present if the background noise of color Doppler appeared as a colored snowstorm across the image. EIT ventilation image: upper right (UR) 8%, upper left (UL) 48%, lower right (LR) 16%, lower left (LL) 27% (% denoted the ventilation distribution portion in each region of interest). Low ventilated regions are marked in dark blue and high ventilated regions in light blue to white. EIT perfusion image: UR 9%, UL 45%, LR 6%, LL 40%. Regions with high perfusion are marked in red and low perfusion in green. EIT *V*-*Q* matching image: percentage of *Shunt%* area in red was 10.34% of the lung regions, *DeadSpace%* area in grey 32.41%, and *VQ* Match% region in yellow 57.24%. Details of the V-Q distribution calculation were described in a previous study [5]. PEEP = 14 cmH_2_O After lung recruitment and drainage of pleural effusion, from left to right ultrasound: demonstrated decreased pleural effusion and recruited consolidated lung tissue in the right lower lobe. The tissue like pattern disappeared, and the corresponding region appeared subpleural debris and B line. Color Doppler ultrasound also showed the absent vascularity. EIT ventilation image: UR 18%, UL 20%, LR 23%, LL 38%. EIT perfusion image: UR 15%, UL 57%, LR 9%, LL 20%. EIT *V*-*Q* matching image: *Shunt%* 19.23%, *DeadSpace%* 45.13%, and *VQ* Match% 35.64%. The white spots (red arrow) moves synchronously with tidal ventilation indicates dynamic air-bronchogram. P = pleural effusion, Lu = lung, Li = Liver, D = diaphragm. **B** Regional perfusion, ventilation and intrapulmonary shunt assessed by lung ultrasound and EIT in a S-pattern case. SP (supine): from left to right: Ultrasound: demonstrated pleural effusion and tissue-like pattern consolidation in the right (R) lower lobe and the corresponding region on color-mode all visualized two parallel coarse blood flows (white arrows). Red: Pulmonary vessels with blood flow moving towards the probe. Blue: Pulmonary vessels with blood flow moving away from the probe. EIT ventilation image: UR 29%, UL 37%, LR 9%, LL 25%. Perfusion image: UR 11%, UL 17%, LR 30%, LL 43%. *V/Q* match image: *Shunt%* 18.53%, *DeadSpace%* 26.14%, and *V/Q* Match% 55.33%. PP (prone positioning): from left to right, ultrasound: demonstrated decreased pleural effusion and recruited consolidated lung tissue in the right lower lobe, and color Doppler ultrasound also showed the decreased vascularity. EIT ventilation image: UR 31%, UL 13%, LR 26%, LL 30%. EIT perfusion image: UR 22%, UL 13%, LR 33%, LL 32%. EIT V-Q matching image: Shunt% 14.81%, DeadSpace% 11.78%, and VQ Match% 73.40%

Not only pulmonary artery embolism but also pulmonary capillaries compression and micro-embolism could cause a perfusion defect in the lung consolidation. COVID-19 also has a high dead-space percentage, which is similar to D-pattern [3]. It might be helpful to determine the location of blood obstruction in the consolidation by assessing pulmonary vessel by color Doppler. Here, we stressed only use ultrasound to associate lung consolidation with dead-space(D pattern) is insufficient, and combined EIT is recommended.

2. Shunt pattern (S-pattern) was defined as a well or relatively normal perfusion with severe impaired ventilation that result in a regional shunt in the related region of lung consolidation. In EIT image, high regional Shunt% is observed, whereas in ultrasound, pulsatile tree-like, tortuous or homogeneously distributed fragmented vascular structures could be identified through several respiratory cycles in any part of the consolidated tissue [2]. Potential pathophysiologic mechanisms for the S-pattern are dysfunction of hypoxic pulmonary vasoconstriction, regional vessels dilation by inflammation. Intrapulmonary shunt has been taken as an important impact on oxygenation in the lung consolidation. With aim to improve regional V-Q matching, improving lung aeration and redistributed lung blood flow (recruitment maneuver, prone positioning) are important in the S-pattern.

To demonstrate the idea, a clinical case of S-pattern was presented. A 55-year-old man, who received the aortic valve replacement due to the severe aortic insufficiency, suffered from refractory hypoxemia on the first postoperative day. The ventilation defect was worse than perfusion defect, which result in intrapulmonary shunt in dependent regions (Fig. 1B). Moreover, pleural effusion and massive tissue-like pattern consolidation were found in the dependent regions. The color Doppler found two parallel vascular flows in the centric part of the consolidated tissue, which also indicate intrapulmonary shunting in dependent regions [1]. After prone positioning for 17 h, the V/Q match was significantly improved.

To our best knowledge, this is the first clinical report combined ultrasound and electrical impedance tomography for categorizing regional perfusion in lung consolidation. The compute and incorporate the anatomical dead-space and cardiac output could provide EIT map of regional ventilation-perfusion matching [4]. Ultrasound has advantage of non-invasive cardiac output monitoring at the bedside, which further combine with EIT for more accurate regional V/Q mapping. There might be gray zone between S pattern and D pattern. Further studies are required to determine the specific parameters and cutoff value for the two typical patterns.

## Data Availability

Not applicable.

## Abbreviations

RM: Recruitment maneuvers
EIT: Electrical impedance tomography
CTPA: Computed tomography pulmonary angiography
SP: Supine
PP: Prone positioning

## References

[1] Mongodi S, Bouhemad B, Iotti GA, Mojoli F (2016). An ultrasonographic sign of intrapulmonary shunt. Intensive Care Med.

[2] Haaksma ME, Smit JM, Heldeweg MLA, Nooitgedacht JS, de Grooth HJ, Jonkman AH, Girbes ARJ, Heunks L, Tuinman PR (2022). Extended lung ultrasound to differentiate between pneumonia and atelectasis in critically ill patients: a diagnostic accuracy study. Crit Care Med.

[3] Gattinoni L, Chiumello D, Caironi P, Busana M, Romitti F, Brazzi L, Camporota L (2020). COVID-19 pneumonia: Different respiratory treatments for different phenotypes?. Intensive Care Med.

[4] Borges JB, Alcala GC, Mlček M (2020). A step forward towards a bedside and timely monitoring of regional ventilation/perfusion matching. Am J Resp Crit Care Med.

[5] He H, Chi Y, Long Y, Yuan S, Zhang R, Yang Y, Frerichs I, Moller K, Fu F, Zhao Z (2021). Three broad classifications of acute respiratory failure etiologies based on regional ventilation and perfusion by electrical impedance tomography: a hypothesis-generating study. Ann Intensive Care.

